# Correction to “Identification and Functional Analysis of a Novel *NSD2* Missense Variant in a Patient With Rauch‐Steindl Syndrome”

**DOI:** 10.1002/mgg3.70252

**Published:** 2026-06-14

Xu, S. , G. Li , Y. He , et al. 2026. “Identification and Functional Analysis of a Novel *NSD2* Missense Variant in a Patient With Rauch‐Steindl Syndrome.” Molecular Genetics & Genomic Medicine 14, no. 6: e70238. 10.1002/mgg3.70238.42216445 PMC13238879

In the published version of the above article, the second author's name was misspelled. The correct name is **Guoqiang Li**.

We apologize for this error.

